# Commencement Exercises

**Published:** 1891-07

**Authors:** 


### COMMENCEMENTS.

### Southern Medical College—Dental Department.

The fourth annual commencement exercises of the Dental Department of the Southern Medical College were held at Concordia Hall, Atlanta, Ga., on Saturday evening, February 28, 1891.

The valedictory was delivered by W. E. Speir, D.D.S., and the annual address by Mr. Hamilton Douglas.

The number of matriculates for the session was one hundred and five.

The degree of D.D.S. was conferred on the following graduates by Dr. T. S. Powell, President of the Board of Trustees:

| NAME. | RESIDENCE. | NAME. | RESIDENCE. |
|---|---|---|---|
| E. G. E. Anderson, A. B. | Tennessee | N. S. Lea | South Carolina |
| H. J. Arnold | Georgia | D. S. Lightcap | Alabama |
| F. L. Adams | Alabama | D. R. Lide | Georgia |
| J. K. Blasingame | Georgia | W. A. Lane | Alabama |
| S. J. Bivings | South Carolina | H. W. Lubben | Texas |
| W. O. Breedlove | Alabama | R. S. McArthur | Georgia |
| W. E. Beacham | Georgia | J. H. Meritt | Georgia |
| J. B. Bearden, M.D | Georgia | J. B. Moncrief | Georgia |
| E. W. Clark | Georgia | F. J. Pulford | Louisiana |
| G. W. Carreker | Georgia | W. R. Pearson | Georgia |
| J. B. Dorsett | Georgia | J. A. Reed | Georgia |
| B. C. Duncan | Alabama | J. H. Rush | Missssippi |
| E. G. Griffin | Georgia | W. E. Speir | Georgia |
| L. D. Gale | Georgia | C. L. Toole | Georgia |
| T. L. Greene | Alabama | W. J. Wade | Georgia |
| E. L. Hanes | Georgia | J. M. Wilkes, M.D | Georgia |
| T. P. Hinman | Georgia | H. R. Williams | Georgia |
| D. H. Harris | Georgia | J. W. Wade | New York |
| I. S. Harn | Georgia | J. E. Woodward | Georgia |

### University of Maryland—Dental Department.

The annual commencement exercises of the Department of Dental Surgery of the University of Maryland were held at the Academy of Music, Baltimore, Md., on Wednesday, March 18, 1891.

The mandamus was read by the Dean, Ferdinand J. S. Gorgas, M.D., D.D.S.; the address to the graduates was delivered by Rev. J. J. G. Webster, D.D., and the class oration by William H. Connor, D.D.S.

The number of matriculates for the session was one hundred and sixty-three.

The degree of D.D.S. was conferred on the following graduates by the Dean, Dr. Gorgas, in the absence, from illness, of Hon. S. Teackle Wallis, L.L.D., Provost of the university:

| NAME. | RESIDENCE. | NAME. | RESIDENCE. |
|---|---|---|---|
| Harry W. Allwine | Nebraska | John C. Loeschcke | Germany |
| J. Perrin Anderson | South Carolina | E. A. Martin, L.D.S | Canada |
| H. C. Bagby, M.D | California | Oscar Matt | New York |
| Denison H. Baldwin | Virginia | Virgil J. McComb | Missouri |
| Frederick A. Barr | Montana | C. M. McKelvey | Pennsylvania |
| James G. Benjamin | Montana | T. Benton Moore | Pennsylvania |
| Capers W. Blalock | Florida | F. A. Pattinson | New York |
| John D. Booth | Canada | William B. Poist | District Columbia |
| J. W. Boozer, A.B | South Carolina | Wm. E. Prather | North Carolina |
| Aubrey R. Bowles | Virginia | Bernard F. Riedel | Maryland |
| Emory M. Bowlus | Maryland | Richie W. Riley | South Carolina |
| Charles S. Boyette | North Carolina | Johannes Rilke | Germany |
| Elvie S. Boyle | Maryland | F. Robinson, L.D.S | England |
| S. S. Brotherton | Iowa | Geo. B. Rounds | Canada |
| George L. Bruce | Maryland | H. Sengebusch | Germany |
| F. W. Chessrown | Pennsylvania | Henry D. Snow | Maryland |
| M. H. P. Clark | North Carolina | E. A. Solomons | South Carolina |
| Wm. H. Connor | New Jersey | Alfred E. Sparks | Canada |
| G. L. Deichmann | Maryland | A. H. Sprinkel | Virginia |
| William Earle | South Carolina | C. C. Sprinkel | Virginia |
| Will H. Ewald | Virginia | F. Myron St. John | Connecticut |
| Wm. M. Feild | Virginia | F. P. Stehley | West Virginia |
| Herbert F. Gorgas | Maryland | J. F. Stevens | Pennsylvania |
| J. William Grove | Pennsylvania | G. W. Stevenson | New York |
| W. Oakley Haines | Maryland | R. E. L. Taliaferro | Virginia |
| Jake V. Haller | Virginia | C. A. Turner | Canada |
| Henry F. Harris | Virginia | A. W. A. Volck | Germany |
| Marion Y. Hart | Virginia | Albert S. Wells | North Carolina |
| Will W. Hayes | Pennsylvania | George F. White | New York |
| J. Henry Hoffman | Maryland | R. J. Whitfield | Canada |
| Charles W. Howard | New Hampshire | C. E. Wingo, M.D | Maryland |
| Edward T. Jones | Virginia | Charles R. Wood | New Hampshire |

### Western Dental College of Kansas City.

The first annual commencement exercises of the Western Dental College of Kansas City were held in the Music Hall, Kansas City, Mo., on Friday, March 13, 1891.

The faculty address was delivered by Professor H. S. Lowry; an address was delivered by Rev. S. S. Laws, and the valedictory by J. H. Cromwell, D.D.S.

The number of matriculates for the session was sixty-two.

The degree of D.D.S. was conferred on the following graduates by Professor D. J. McMillen, dean:

| NAME. | RESIDENCE. | NAME. | RESIDENCE. |
|---|---|---|---|
| S. S. Brown | Missouri | H. B. Lowry | Ohio |
| J. H. Comwell | Missouri | Frank Nelson | Kansas |
| B. T. Edmiston | Arkansas | J. D. Roy | Missouri |
| H. B. Heckler | Ohio | C. J. Sawyer | Kansas |
| C. W. Lukens | Oregon | | |

### Chicago College of Dental Surgery.

The ninth annual commencement exercises of the Chicago College of Dental Surgery (Dental Department of the Lake Forest University) were held at the Columbia Theatre, Chicago, Ill., on Tuesday, March 24, 1891, at 2:30 p.m.

The annual address was delivered by W. C. Roberts, D.D., LL.D., president of the university, and the doctorate address by Calvin S. Case, M.D., D.D.S.

The number of matriculates for the session was three hundred and twenty-three.

The degree of D.D.S. was conferred on the following graduates by Truman W. Brophy, M.D., D D.S., president of the college:

| NAME. | RESIDENCE. |
|---|---|
| Charles Gant Adams | Illinois |
| Frank C. Allen | Indiana |
| Evan Bailey | Illinois |
| Claude G. Baker | Illinois |
| William H. Balluff | Illinois |
| Horace H. Ball | Illinois |
| Charles S. Bigelow | Florida |
| *F. H. Birchmeier | Illinois |
| Charles H. Boughton | Illinois |
| David A. Bowerman | Canada |
| Raymond W. Boyer | Michigan |
| Francis M. Bozer | Indiana |
| Clare S. Bradley | Wisconsin |
| Oscar F. Brightfield | Pennsylvania |
| *John B. Burns | Illinois |
| J. W. Cameron, M.D | Wisconsin |
| Frank Chaffee, M.D | Indiana |
| James E. Clark | Iowa |
| D. F. Cotterman | Indiana |
| George M. Crisup | Illinois |
| *Albert M. Davis | Michigan |
| Henry F. Dean | Wisconsin |
| Fred. C. Devendorf | Wisconsin |
| Wesley G. DeVore | Illinois |
| Willis H. Dwight | Iowa |
| David H. Evey | Illinois |
| W. H. Fancher, M.D | Wisconsin |
| *Manfred S. Fraser | Colorado |
| Donald McK. Gallie | Canada |
| Henry I. Gibson | Wisconsin |
| Winthrop Girling | Illinois |
| Walter J. Godfrey | Illinois |
| John J. Grout | Iowa |
| Hans A. Guenther | Illinois |
| Joseph E. Hart | Illinois |
| A. A. H. Hamer | Holland |
| James E. Harned | Illinois |
| George E. Hawkins | Illinois |
| George E. Henry | Illinois |
| *Charles F. Hunt | Illinois |
| Edgar C. Kaye | Illinois |
| Edward F. Keefe | Illinois |
| Charles G. Keehn | Indiana |
| S. De Bruce Knapp | Wisconsin |
| *Elmore D. Lyon | Illinois |
| James F. Martin | South Dakota |
| C. A. McDermand | Canada |
| Fred R. McLean | Illinois |
| Hugh McNeil | Michigan |
| Will F. Michaelis | Illinois |
| *Edwin D. Neff. | Illinois |
| Charles Odell | Illinois |
| *Frank A. Paine | Illinois |
| Herman G. Pape | Iowa |
| John I. Parker | Illinois |
| R. McC. Pearce | Illinois |
| Stephen C. Pierce | Wisconsin |
| Frank J. Powell. | Wisconsin |
| Ulysses G. Poyer | Illinois |
| John H. Ramsey | Pennsylvania |
| Edd S. Reed | Illinois |
| William A. Reed | Iowa |
| *William T. Reeves | Illinois |
| *Paul A. Riebe | Wisconsin |
| Mervin B. Rimes | Michigan |
| Warren M. Ringsdorf | Wisconsin |
| Cyrus H. Robinson | Minnesota |
| Archer W. Rodman | Wisconsin |
| Peter H. Ruus | Illinois |
| John M. Saucerman | Illinois |
| Philipp J. V. Schnell | Illinois |
| Colfax Schuyler | Illinois |
| Louis A. Schultz | Illinois |
| William H. Simmons | Wisconsin |
| Howard T. Smith | Illinois |
| Oscar R. Smith | Illinois |
| *Frederick A. Stetson | Illinois |
| William W. Strayer | Ohio |
| *Henry C. Strong | Illinois |
| Sherman T. Taylor | Illinois |
| *Charles N. Thompson | Illinois |
| Peter W. Thorelius | Illinois |
| Frank S. Trickey | Illinois |
| Wallace E. Tucker | Illinois |
| *W. S. Van Nostrand | Illinois |
| Patrick H Welch | Wisconsin |
| Adolph A. Wendell | Wisconsin |
| Raymond J. Wenker | Wisconsin |
| Jarvis W. Wetherbee | Iowa |
| Roy P. Wilcox | Illinois |
| L. S. Wilson, M.D. | Iowa |
| Jonas T. Williams | Iowa |
| Frank V. Yorker | Michigan |
| Clarence W. Young | Michigan |

*Certificate of honor for having attended a spring course of lectures.

### Royal College of Dental Surgeons of Ontario.

The annual commencement exercises of the Royal College of Dental Surgeons of Ontario were held in the Normal School Hall, Toronto, on Tuesday, April 7, 1891, at 8 p.m.

The valedictory was delivered by W. Richardson, L.D.S., and the address to the graduates by J. Taft, M.D., dean of the Dental Department of the University of Michigan.

The number of matriculates for the session was sixty-eight.

The following graduates were admitted Licentiates of Dental Surgery, the certificates being presented by H. T. Wood, M.D.S. president of the board of directors:

H. D. Boyes.

Thos. Coleman.

W. F. Corbett.

O. W. Daly.

S. W. Frith.

C. D. Green.

G. H. Henderson.

J. E. Holmes.

E. R. Howes.

C. W. Lennox, D.D.S.

C. H. Lount.

O. Lillie.

Jas. Letherdale.

G. S. Martin.

A. H. Mabee.

Jas. McBride.

H. S. McLaughlin.

F. R. Porter.

W. Richardson.

J. J. Sinon.

M. J. Sisley.

H. J. Stingle.

H. R. Thornton, D.D.S.

A. T. Watson, D.D.S.

J. E. Wilkinson.

W. R. Winters.

G. F. Wright.

All of the Province of Ontario.

President Wood also conferred the degree of Master of Dental Surgery (M.D.S.) upon—

Thomas Henderson, L.D.S., D.D.S., Toronto.
Orlando H. Ziegler, L.D.S., London.
W. A. Leggo, L.D.S., D.D.S., Ottawa.

### Meharry Dental Department—Central Tennessee College.

The fifth annual commencement exercises of the Meharry Dental Department of Central Tennessee College were held, in connection with those of the medical and pharmaceutical departments, at Masonic Theatre, Nashville, Tenn., on Thursday, February 19, 1891.

The salutatory address was made by A. O. Lockhart, and addresses were also delivered by Dr. N. G. Tucker, Senator Early and others.

The number of matriculates for the session in the dental class was five.

The degree of D.D.S. was conferred by Dr. J. Braden, President of the Faculty, on G. W. Bunn, of Arkansas.

### University of Pennsylvania—Department of Dentistry.

The twelfth annual commencement of the Department of Dentistry of the University of Pennsylvania was held, in connection with that of the medical department, at the American Academy of Music, Philadelphia, Pa., on Friday, May 1, 1891, at 12 o'clock M.

The valedictory address was delivered by James Tyson, M.D., Professor of Clinical Medicine.

The number of matriculates for the session was two hundred and six.

The degree of D.D.S. was conferred on the following graduates by William Pepper, M.D., LL.D., provost of the university:

| NAME. | RESIDENCE. |
|---|---|
| W. Lynn Adamy | New York |
| R. Antoine, M.D | Austria |
| W. T. Arrington, Jr | Tennessee |
| Myron Barlow | Massachusetts |
| Geo. M. C. Barnard | Massachusetts |
| Charles H. Barnes | Pennsylvania |
| A. H. Beers, M.D | Canada |
| H. Boennecken, M.D | Germany |
| Warren E. Booker | Massachusetts |
| Arturo Borja | Mexico |
| Wm. I. Brenizer | Ohio |
| Oswald M. Brown | Illinois |
| James R. Burnett | Illinois |
| Frank L. Caldwell | New York |
| Obe B. Caldwell | Kentucky |
| Elliott R. Carpenter | Michigan |
| H. E. Chesebrough | New York |
| Charles P. Chupein | Pennsylvania |
| H. B. Clearwater | Pennsylvania |
| Arthur Brooks Cox | Australia |
| William H. Cregan | Massachusetts |
| James S. Darragh | Pennsylvania |
| Charles H. Davis | Pennsylvania |
| Charles H. Dilts | New Jersey |
| Aug. H. Dreher | North Carolina |
| Theodore S. Fay | Germany |
| Edward Fetscherin | Switzerland |
| J. Milton Fogg | Pennsylvania |
| George J. Frey | New York |
| C. Franklin Gibbs | Connecticut |
| William J. Giles | Canada |
| Edson M. Green | Pennsylvania |
| Samuel S. Haines | New Jersey |
| William H. Haines | Ohio |
| Karl Hampe | Germany |
| Thomas Holder | New Hampshire |
| Fred. B. Howe | New York |
| Sidney F. Jacobi | Indiana |
| Edward V. Larkin | Pennsylvania |
| Henry M. Laros | Pennsylvania |
| M. W. Levkowicz | Costa Rica |
| Frederic W. McCall | New York |
| J. Atkinson McKee | Pennsylvania |
| Michael Maguire | Pennsylvania |
| J. F. Mayer | Pennsylvania |
| Archibald Miller | Pennsylvania |
| John Henry Muller | Switzerland |
| Frank L. Naramore | South Carolina |
| Patrick J. O'Connor | Pennsylvania |
| Louis E. de la Ossa | U. S. of Col. |
| Richard B. O'Sullivan | Canada |
| Geo. Janvier Paynter | Pennsylvania |
| Alfred H. Porter | Australia |
| Frank C. Porter | England |
| Leon E. Putnam | Pennsylvania |
| Louis E. Rauch | Pennsylvania |
| Charles A. Rice | New Jersey |
| Louie J. Rounds | New York |
| Jacques Russli | Switzerland |
| Earle J. Sallada | Pennsylvania |
| B. F. Sayres | Pennsylvania |
| Ernst Schiffmann | Switzerland |
| Joseph W. Schwacke | South Carolina |
| William A. Siddall | Ohio |
| Fred. A. Smith | New York |
| Julian Smith | New York |
| Wm. C. Speakman | Delaware |
| Louis J. Stephan | Wisconsin |
| Giovanni A. Stoppani | Switzerland |
| Charles H. Tillotson | Illinois |
| Chas. J. Tinkham, Jr | Illinois |
| Wm. B. Townsend | Pennsylvania |
| Robert W. Volk | Massachusetts |
| Clarence V. Watts | Iowa |
| Wm. H. Waugaman | Pennsylvania |
| Theo. H. Whitbeck | New York |
| Elmer B. White | New York |
| C. H. Wilson | Pennsylvania |
| John C. Wiltbank | Delaware |
| Geo. F. Woodbury | Pennsylvania |

DEGREE CONFERRED JUNE 5, 1890.

| | |
|---|---|
| Milton H. Evans | Pennsylvania |
| Clarence M. Root | New York |
| Edward E. Rossbach | Germany |

### Northwestern College of Dental Surgery.

The eighth annual commencement exercises of the Northwestern College of Dental Surgery were held at the college building, 1203 Wabash avenue, Chicago, Ill., on Tuesday evening, March 31, 1891.

The doctorate address was delivered by Professor C. G. B. Klophel, M.D.; the valedictory address by William S. Milligan, D.D.S.

The degree of D.D.S. was conferred on the following graduates by Professor R. W. Clarkson, dean of the college:

Jas. Sampson Goodridge..................England.
George Michael Honlihan..................Wisconsin.
Wm. Savage Milligan.....................Pennsylvania.

The honorary degree of D.D.S. was conferred on Richie DeLan, of Massachusetts.

### United States Dental College.

The first annual commencement exercises of the United States Dental College were held at the Auditorium, Chicago, Ill., Thursday, March 26, 1891.

The opening address was made by Dr. J. J. M. Angear; the valedictory was delivered by H. Barry Millican, D.D.S., and an address was also delivered by Dr. G. Frank Lydston.

The degree of D.D.S. was conferred on the following graduates:

Edwin Burke.

William M. Evans.

Park B. Leason.

H. Barry Millican.

Paul A. Piehl.

Gideon A. Price.

John C. Prill.

F. F. Scherman.

Emil Seghers.

William H. G. White.

F. J. Warrenfells.

### College of Dentistry of the University of Denver.

The third annual commencement exercises of the College of Dentistry of the University of Denver were held, in connection with those of the medical and pharmaceutical departments, at the Trinity M. E. Church, Denver, Col., on Tuesday evening, April 14, 1891.

The annual address was delivered by J. C. Davis, M.D. The charge to the graduating class was given by P. T. Smith, D.D.S., dean.

The number of matriculates for the session was thirteen.

The degree of D.D.S. was conferred on the following graduates by Chancellor McDowell, of the University of Denver :

| | |
|---|---|
| William A. Armstrong......New York | John S. Donaldson............Colorado |
| Robert S. Clarke..... ..........Illinois | Edwin C. Hember.............England |
| James R. Donaldson..... ....Colorado | |

### Baltimore College of Dental Surgery.

The fifty-first annual commencement exercises of the Baltimore College of Dental Surgery were held at Harris' Academy of Music, Baltimore, Md., on Monday evening, March 23, 1891.

The annual oration was delivered by Dr. Wayland Ball, and the valedictory address by Edward Hamm, D.D.S., of the graduating class.

The number of matriculates for the session was two hundred and twenty-four.

The degree of D.D.S. was conferred on the following graduates by Professor M. W. Foster:

| NAME. | RESIDENCE. | NAME. | RESIDENCE. |
|---|---|---|---|
| David L. Aber | Pennsylvania | Robert S. Henry | Maryland |
| Charles E. Altemus | Pennsylvania | Frank W. Hill | Iowa |
| Louis D. Archinard | Louisiana | George H. Jackson | Pennsylvania |
| Walter V. Austin | Minnesota | Walter L. Jones | Mississippi |
| Theodore A. Bailey | Georgia | George Kress | Ohio |
| Calvin D. Brown | California | T. B. Leatherbury | Virginia |
| John H. Bulett | Pennsylvania | Harold S. Lockwood | New York |
| James K. Burgess | Virginia | John C. Maloney | Virginia |
| James F. Butts | West Virginia | E. B. Marshall, Jr | Georgia |
| Joseph J. Battle | North Carolina | G. V. Milholland | Maryland |
| Samuel E. B echer | Pennsylvania | Frank S. Morton | Nova Scotia |
| Horace I. Beemer | New Jersey | James S. McDonald | Pennsylvania |
| Ernest Bent | Massachusetts | William E. Nye | California |
| Robert C. Bradshaw | Maryland | Cameron E. Orndorf | Pennsylvania |
| John J. Carroll | West Virginia | Edward B. Parker | Virginia |
| Frank M. Conkey | Illinois | Oswald A. Parker | Nova Scotia |
| Willard L. Chapin | Pennsylvania | John C. Pfeiffer | Maryland |
| Calvin O. Chunn | Florida | Mozart W. Rainold | Louisiana |
| L. M. Cleckley | Georgia | Herman Reichhelm | Germany |
| Charles G. Colby | Connecticut | Charles P. Rice | Pennsylvania |
| Thomas A. Cronin | Maryland | George O. Roberts | Germany |
| Ulysses A. Dalton | New York | Fred. C. Royce | New York |
| John W. David | Texas | G. P. Schumacker | Massachusetts |
| Ernest C. Deuel | California | Thomas W. Sharpe | Pennsylvania |
| James F. Downs | Maryland | Warren M. Sharp | New Brunswick |
| William Dick | California | Otis F. Sims | Florida |
| Edward Eggleston | Virginia | W. M. Steinmeyer | South Carolina |
| Josiah G. Fife | Texas | Wm. M. Stewart | New York |
| James G. Findlay | Ontario | George E. Stoddard | Vermont |
| Branch Garner | Louisiana | Charles B. Tarr | Maine |
| James B. Gould | Virginia | W. E. Teaseley | Virginia |
| William F. Graham | South Carolina | Thomas K. Tharp | Georgia |
| Harvey T. Greenlaw | New Brunswick | Rowland H. Walker | Virginia |
| Guy Gress | Pennsylvania | Frederick R. Wilder | Vermont |
| Edward Hamm | Massachusetts | Frank C. Wilson | Georgia |
| Lewis A. Hauser | North Carolina | Herrmann Wurzel | Germany |
| Samuel J. Heindel | Pennsylvania | W. E. Wolfrum | Wisconsin |
| Elmer E. Henry | Pennsylvania | Andrew Youngs | New York |

### Missouri Dental College.

The twenty-fifth annual commencement exercises of the Missouri Dental College were held, in connection with those of the St. Louis Medical College, at Memorial Hall, St. Louis, Mo., on Thursday, March 12, 1891.

The address to the class was delivered by Professor George Homan and the prizes presented by Dr. Wm. N. Morrison.

The number of matriculates for the session was ninety.

The degree of D.D.S. was conferred on the following graduates by Dr. H. H. Mudd, Dean of the Faculty:

| NAME. | RESIDENCE. |
|---|---|
| Edward L. Beatie | Missouri |
| Arthur C. Bedford | Missouri |
| Moses C. Boswell | Missouri |
| C. L. Brudewold, M.D. | Pennsylvania |
| James S. Coyle | |
| Marvin L. Cumming | Illinois |
| Charles E. Dungan | Illinois |
| Walter R. Eckle | Missouri |
| James B. Harrison | Indiana |
| George D. Kennedy | Colorado |
| John W. Markwell | Indiana |
| John E. Masterson | Texas |
| Joseph De G. Peck | Kansas |
| Reuben G. Porter | Michigan |
| Arthur G. Purdy | Illinois |
| Wm. T. Rutledge | Missouri |
| Herman Saxenmeyer | Illinois |
| Herman O. Settelon | Switzerland |
| Samuel Schrantz | Missouri |
| Carl E. Schumacher | Westphalia |
| George C. Schwarz | Illinois |
| John O. H. Thiele | Germany |
| Hugh G. Voorhies | Illinois |
| James F. Wallace | Missouri |
| Matthew D. Wilson | Missouri |
| Park H. Winans | Illinois |

### Tennessee Medical College—Dental Department.

The second annual commencement exercises of the Department of Dentistry of the Tennessee Medical College were held at Staub's Theatre, Knoxville, Tenn., on the evening of March 19, 1891.

An address was delivered by Rev. R. R. Sutherland, D.D., and the charge to the graduating class was given by Professor Charles M. Drake.

The number of matriculates for the session was eighteen.

The degree of D.D.S. was conferred on the following graduates by Colonel E. J. Sanford, President of the Board of Trustees:

| NAME. | RESIDENCE. |
|---|---|
| R. S. Booth | North Carolina |
| D. D. Foley | Kentucky |
| H. F. Henderson | Virginia |
| J. A. Keener | Tennessee |
| J. K. Moose | North Carolina |
| T. B. McBride | Pennsylvania |
| Wexler Smathers | North Carolina |

### College of Dental Surgery of the University of Michigan.

Tae sixteenth annual commencement of this department was held in University Hall, Ann Arbor, Thursday, June 25th, 1891. The number of matriculates was one hundred and thirty-two.

The annual commencement address to the graduates of the university was delivered by the Hon. J. C. Gilman, of Johns Hopkins University, Baltimore.

The degree of Doctor of Dental Surgery was conferred on the following candidates by the president of the university, James B. Angell, L.L.D.:

Walter Horace Booth.

James Frank Cook.

Manuel Vicente del Valle.

Rokus Christian Devries.

Arthur Aaron Deyoe.

Frank Chester Dorrance.

Charles Henry Edwards.

Frederick William Fleming.

Walter Jesse Green.

Frank Snydey Henry.

William Edward Kearns.

Gordon Grant McCoy.

Austin McGuire.

Clinton Floyd Metcalf.

Arthur Werner Mueller.

Pascal Pratt Nelson.

Charles Sigfried Rudolph Osius.

Michael More Park.

Wilsie David Reed.

Clinton Robert Scott.

Alfred Louis Sickler.

Charles Perce Stone.

Jonathan Ray Taft.

Lewis Carlisle Thayer.

Victor Emanuel Tuttle.

Eldon Waterloo.

Lucy Kate Waterloo.

William Williams, M.D.

Burt G. Winans.

### German-American Dental College.

The annual commencement exercises of the German-American Dental College (Deutsdh-Amerikanische Schule fur Zahnheilkunde) were held in the Grand Palace Hotel Banquet Hall, Chicago, Ill., on Wednesday, March 11, 1891, at 8 P. M.

The address for the faculty was delivered by Prof. Dr. Fritz Brunhoff, who was also the recipient of an honorary degree. The valedictory was delivered by Dr. Felix Arendt, of Russia.

The number of matriculates for the session was twenty-two.

Degrees were conferred on the following graduates by Dr. J. Bernauer, Professor of Histology and Surgery :

| NAME. | RESIDENCE. |
|---|---|
| Felix Arendt | Russia |
| Max Benedikt | Austria |
| P. Krause | Germany |
| Max Kalbe | Germany |
| E. Klemich | Germany |
| M. Muehlhauser | Germany |
| Eugen Mueller | Switzerland |
| Edwin Schenk | Germany |
| C. Schuhmann | Germany |
| C. Schmidt | Germany |
| August Vogele | Germany |

### Indiana Dental College.

The twelfth annual commencement exercises of the Indiana Dental College were held in the Propylæum Building, Indianapolis, Ind., February 27, 1891.

The annual address was delivered by Dr. M. F. Ault, of Kokomo.

The number of matriculates for the session was ninety-six.

The degree of D.D.S. was conferred on the following graduates by Seneca B. Brown, M.D., D.D.S., President of the College:

| NAME. | NAME. |
|---|---|
| A. L. Austin | L. Lichtenwater |
| W. E. Armstrong | Miss L. B. McCollum |
| W. S. Beazley | A. O. McCutcheon |
| J. W. Brimacombe | O. F. Overstreet |
| M. M. Cook | O. Palmer |
| B. D. Curtis | B. T. Perkins |
| C. P. Danks | M. Raschig |
| C. Feigel | G. S. Rhea |
| E. D. Foulds | L. W. Roe |
| C. B. Hayford | P. A. Row |
| H. C. Heaton | Mrs. H. G. Scott |
| C. G. Hoover | E. F. Shields |
| J. B. Jacques | O. V. Simmerman |
| W. A. Johnson | F. Smith |
| M. W. Johnston | M. Smith |
| G. C. Keel | L. J. Stiver |
| M. J. Keightly | C. P. Tinkham |
| O. A. Keiser | C. E. Whitesides |
| R. H. Kiser | C. F. Williams |
| J. M. Lewis | H. M. Zehrung |

### The Deodorization of Iodoform by Creolin.

Dr. Ludwig Vaczi, a practitioner in Nagy-Karoly, communicates to the *Medicidisch-chirurgische Rundschau* his discovery of the power of creolin to deodorize iodoform. He had prescribed an ointment consisting of one part of creolin, two of iodoform and twenty-five parts of vaseline. On the following day he was surprised that not only was the usual color of iodoform ointment changed, but that there was no smell of iodoform and only a slight smell of creolin. He points out how important it is in many cases that the presence of iodoform should not be known by its odor, and considers creolin the very best of all deodorizing drugs for the same. It not only does not irritate, but it is also itself a good disinfectant.—*Lancet.*

### Kansas City Dental College.

The ninth annual commencement exercises of the Kansas City Dental College were held in Grand Avenue M. E. Church, Kansas City, Mo., on Tuesday evening, March 10, 1891.

The faculty address was delivered by Professor W. T. Stark, and an address was also delivered by W. S. Cowherd.

The number of matriculates for the session was one hundred and seven.

The degree of D.D.S. was conferred on the following graduates by C. B. Hewitt, D.D.S., President of the Faculty:

| NAME. | RESIDENCE. |
|---|---|
| Wallar B. Austin | Missouri |
| William K. Aitken | Kansas |
| Alba L. Ashby | Kansas |
| Henry E. Baxter | Kentucky |
| W. C. K. Buchanan | Missouri |
| William P. Baker | Kansas |
| Frederic G. Corey | Kansas |
| Alonzo T. Crow | Kansas |
| Frank J. Claypool | Kansas |
| Archie M. Detrick | Nebraska |
| Charles A. Draper | Missouri |
| Rezin T. Fowler | Missouri |
| Robert P. Greenlee | Missouri |
| Elbert Q. Gibson | Missouri |
| James Galloway | Colorado |
| Harry D. Hines | Iowa |
| F. G. Hunsicker | Kansas |
| William B. Hale | Missouri |
| Aaron L. Hitchins | Kansas |
| John D. V. Kice | Missouri |
| Augustine H. Kirby | Kansas |
| James T. Kenney | Missouri |
| Frank C. Kenney | Missouri |
| Samuel W. Kincaid | Kansas |
| Paul J. Laws | Kansas |
| Charles B. Lyon | Kansas |
| George L. Lewis | Kansas |
| Alfred H. Mann | Kansas |
| William B. Myers | Kansas |
| James R. McLeland | Kansas |
| Wm. H. Ockerman | Missouri |
| Simpson Ockerman | Iowa |
| Arthur D. Park | Missouri |
| Chester B. Reed | Kansas |
| Harry E. Roberts | Illinois |
| Abert O. Sage | Kansas |
| Matt. F. Toler | Kansas |
| Winslow P. Upton | Kansas |
| Henry A. Whitmer | Missouri |
| William N. West | Kansas |
| George H. Woods | Kansas |
| Frank M. Wilson | Iowa |
| Oakley R. Wibking | Kansas |

### How to Wash the Face.

You all, no doubt, think you know how to wash your face, yet many persons have paid two guineas to the distinguished English physician, Sir Erasmus Wilson, for the following advice:

"Fill your basin about two-thirds full of fresh water, dip your face in the water then your hands. Soap the hands well and pass them with gentle friction over the whole face, then dip the face in the water a second time and rinse it thoroughly. A second basin ready with fresh water is a valuable addition. Rain or distilled water, owing to its purity and softness, is the best for washing the face."

